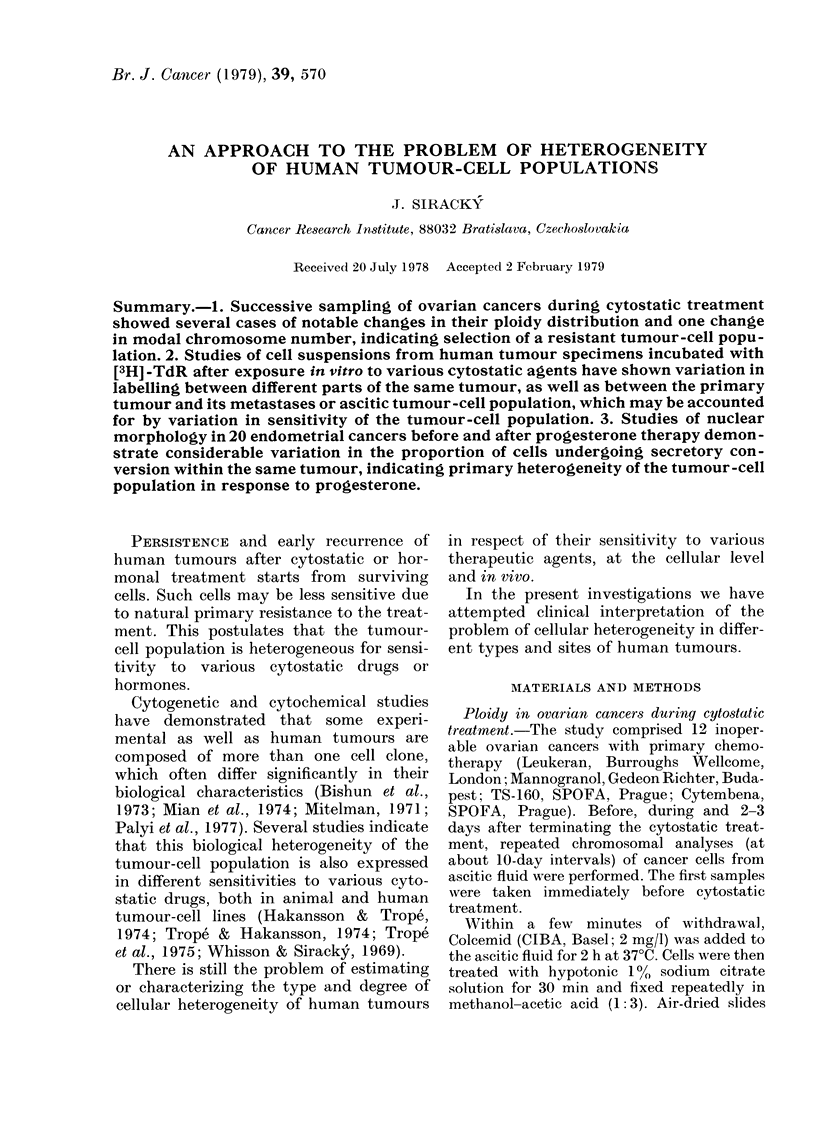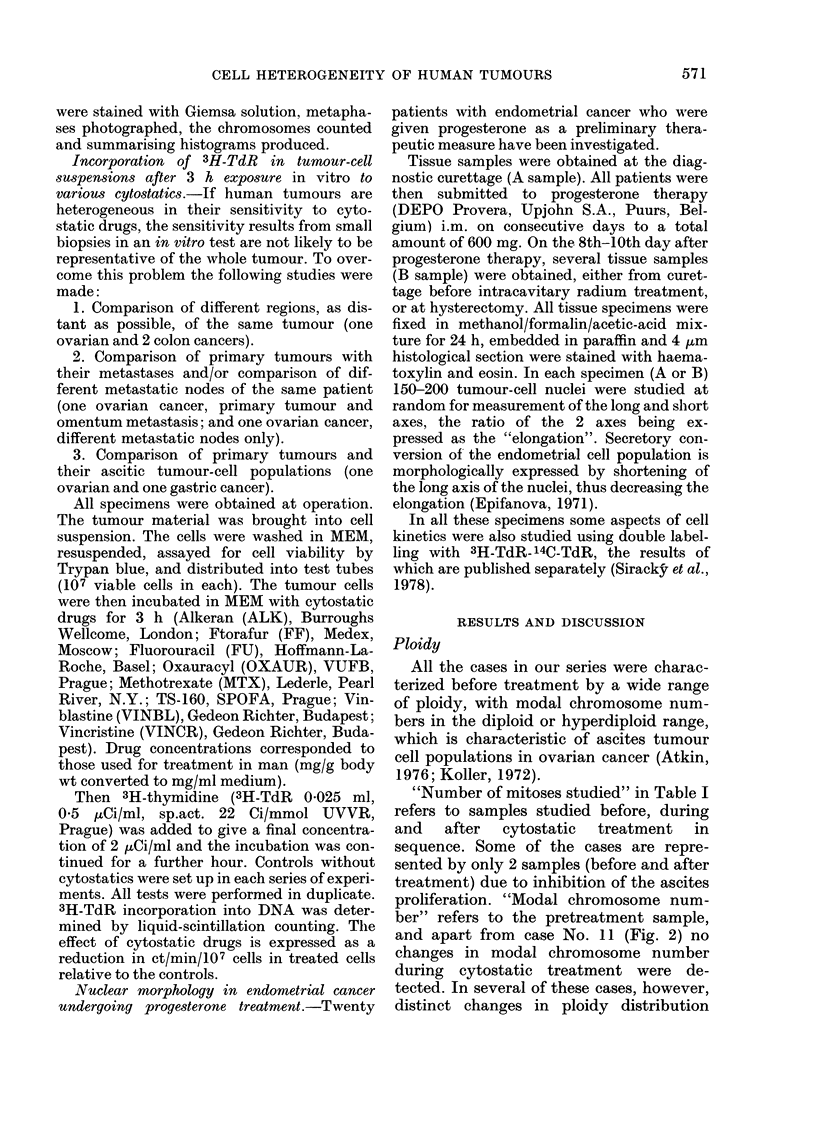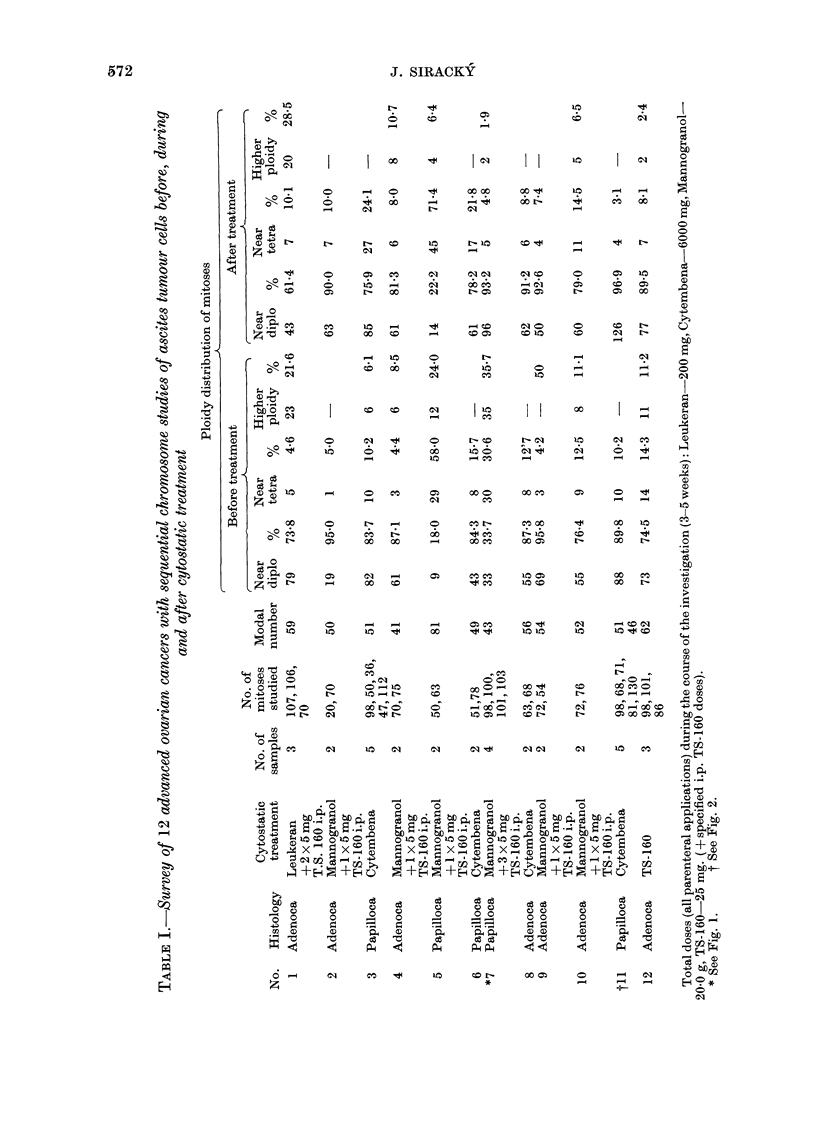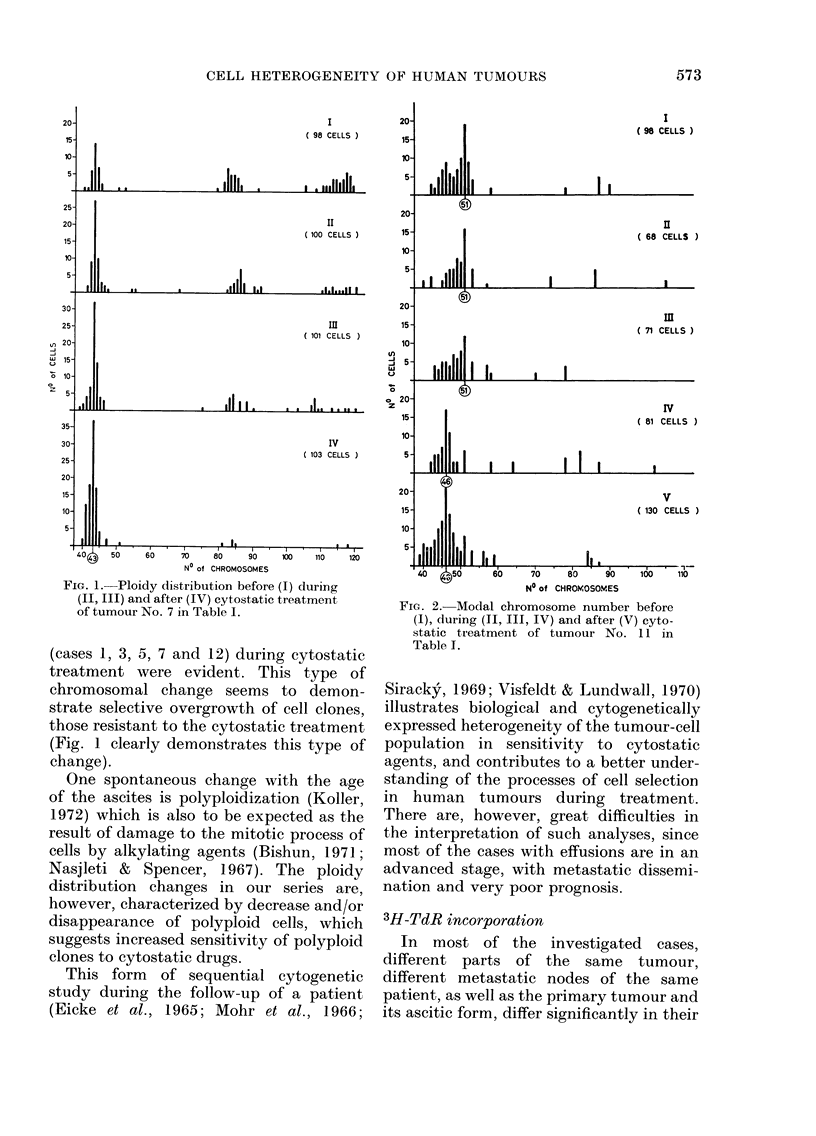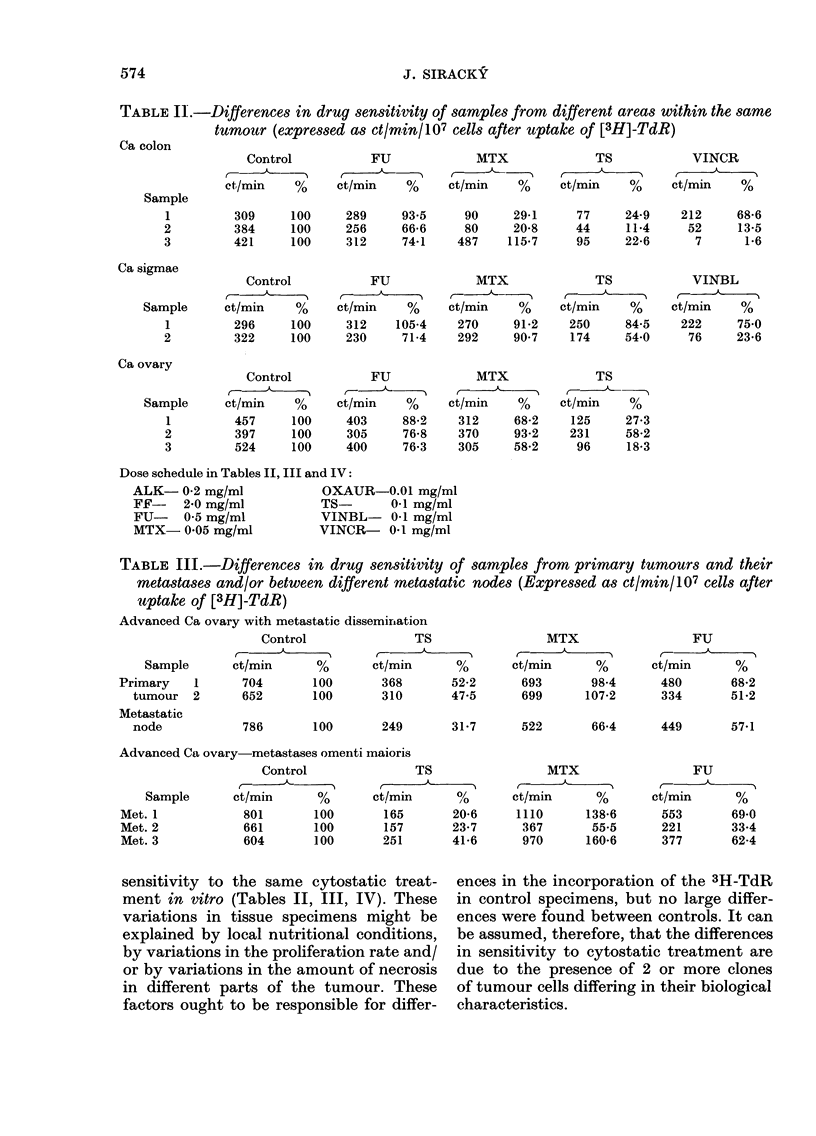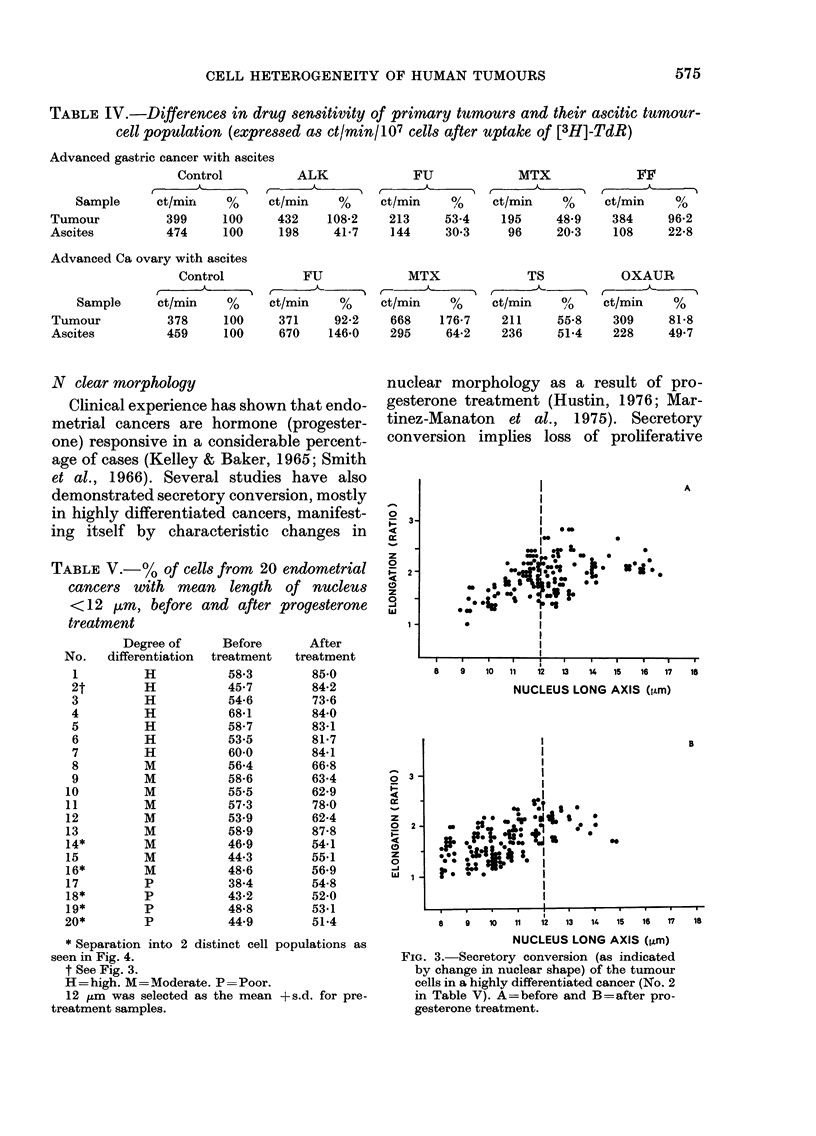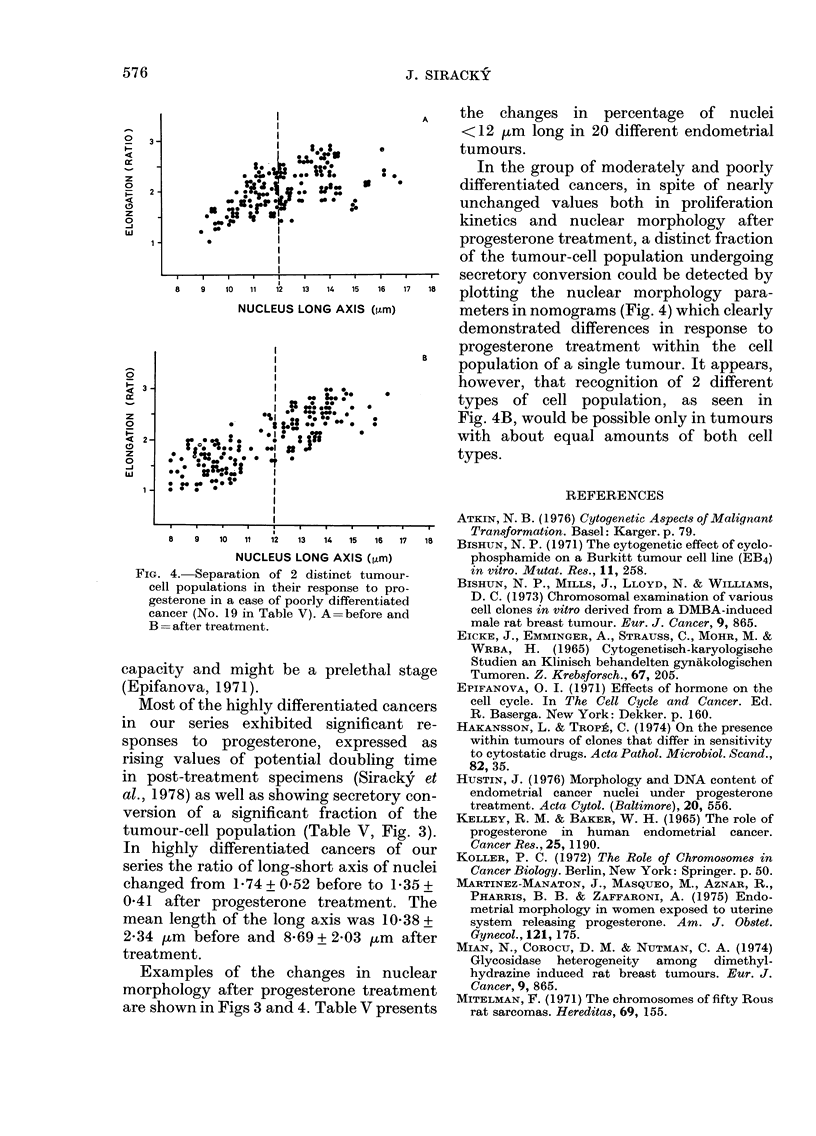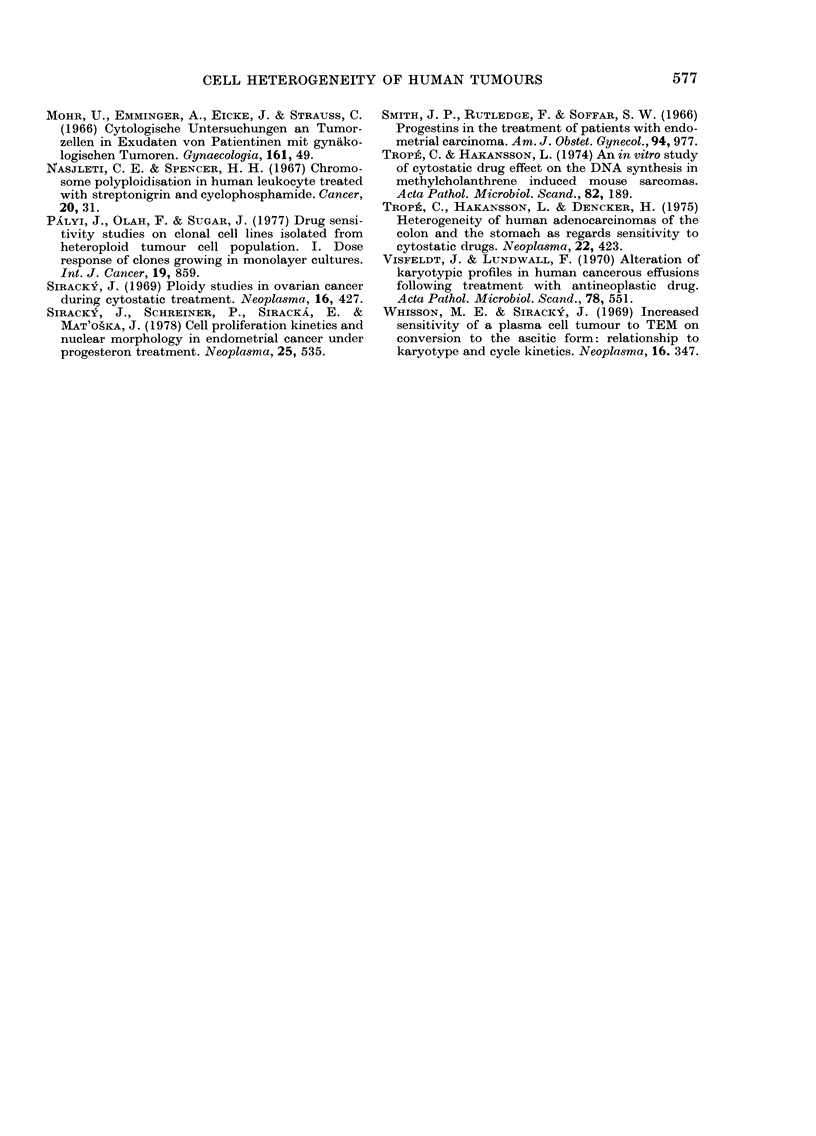# An approach to the problem of heterogeneity of human tumour-cell populations.

**DOI:** 10.1038/bjc.1979.103

**Published:** 1979-05

**Authors:** J. Sirachý

## Abstract

1. Successive sampling of ovarian cancers during cytostatic treatment showed several cases of notable changes in their ploidy distribution and one change in model chromosome number, indicating selection of a resistant tumour-cell population. 2. Studies of cell suspensions from human tumour specimens incubated with [3H]-TdR after exposure in vitro to various cytostatic agents have shown variation in labelling between different parts of the same tumour, as well as between the primary tumour and its metastases or ascitic tumour-cell population, which may be accounted for by variation in sensitivity of the tumour-cell population. 3. Studies of nuclear morphology in 20 endometrial cancers before and after progesterone therapy demonstrate considerable variation in the proportion of cells undergoing secretory conversion within the same tumour, indicating primary heterogeneity of the tumour-cell population in response to progesterone.


*Br. J. Cancer* (1979), **39**, 570

### AN APPROACH TO THE PROBLEM OF HETEROGENEITY OF HUMAN TUMOUR-CELL POPULATIONS

J. SIRACKÝ

*Cancer Research Institute*, 88032 *Bratislava, Czechoslovakia*

Received 20 July 1978 Accepted 2 February 1979

**Summary.—1. Successive sampling of ovarian cancers during cytostatic treatment showed several cases of notable changes in their ploidy distribution and one change in modal chromosome number, indicating selection of a resistant tumour-cell population. 2. Studies of cell suspensions from human tumour specimens incubated with [$^3$H]-TdR after exposure *in vitro* to various cytostatic agents have shown variation in labelling between different parts of the same tumour, as well as between the primary tumour and its metastases or ascitic tumour-cell population, which may be accounted for by variation in sensitivity of the tumour-cell population. 3. Studies of nuclear morphology in 20 endometrial cancers before and after progesterone therapy demonstrate considerable variation in the proportion of cells undergoing secretory conversion within the same tumour, indicating primary heterogeneity of the tumour-cell population in response to progesterone.**

PERSISTENCE and early recurrence of human tumours after cytostatic or hormonal treatment starts from surviving cells. Such cells may be less sensitive due to natural primary resistance to the treatment. This postulates that the tumour-cell population is heterogeneous for sensitivity to various cytostatic drugs or hormones.

Cytogenetic and cytochemical studies have demonstrated that some experimental as well as human tumours are composed of more than one cell clone, which often differ significantly in their biological characteristics (Bishun *et al.*, 1973; Mian *et al.*, 1974; Mitelman, 1971; Palyi *et al.*, 1977). Several studies indicate that this biological heterogeneity of the tumour-cell population is also expressed in different sensitivities to various cytostatic drugs, both in animal and human tumour-cell lines (Hakansson & Tropé, 1974; Tropé & Hakansson, 1974; Tropé *et al.*, 1975; Whisson & Siracký, 1969).

There is still the problem of estimating or characterizing the type and degree of cellular heterogeneity of human tumours in respect of their sensitivity to various therapeutic agents, at the cellular level and *in vivo*.

In the present investigations we have attempted clinical interpretation of the problem of cellular heterogeneity in different types and sites of human tumours.

### MATERIALS AND METHODS

*Ploidy in ovarian cancers during cytostatic treatment*.—The study comprised 12 inoperable ovarian cancers with primary chemotherapy (Leukeran, Burroughs Wellcome, London; Mannogranol, Gedeon Richter, Budapest; TS-160, SPOFA, Prague; Cytembena, SPOFA, Prague). Before, during and 2–3 days after terminating the cytostatic treatment, repeated chromosomal analyses (at about 10-day intervals) of cancer cells from ascitic fluid were performed. The first samples were taken immediately before cytostatic treatment.

Within a few minutes of withdrawal, Colcemid (CIBA, Basel; 2 mg/l) was added to the ascitic fluid for 2 h at 37°C. Cells were then treated with hypotonic 1% sodium citrate solution for 30 min and fixed repeatedly in methanol–acetic acid (1:3). Air-dried slides were stained with Giemsa solution, metaphases photographed, the chromosomes counted and summarising histograms produced.

*Incorporation of $^3$H-TdR in tumour-cell suspensions after 3 h exposure* in vitro *to various cytostatics.*—If human tumours are heterogeneous in their sensitivity to cytostatic drugs, the sensitivity results from small biopsies in an *in vitro* test are not likely to be representative of the whole tumour. To overcome this problem the following studies were made:

1. Comparison of different regions, as distant as possible, of the same tumour (one ovarian and 2 colon cancers).
2. Comparison of primary tumours with their metastases and/or comparison of different metastatic nodes of the same patient (one ovarian cancer, primary tumour and omentum metastasis; and one ovarian cancer, different metastatic nodes only).
3. Comparison of primary tumours and their ascitic tumour-cell populations (one ovarian and one gastric cancer).

All specimens were obtained at operation. The tumour material was brought into cell suspension. The cells were washed in MEM, resuspended, assayed for cell viability by Trypan blue, and distributed into test tubes ($10^7$ viable cells in each). The tumour cells were then incubated in MEM with cytostatic drugs for 3 h (Alkeran (ALK), Burroughs Wellcome, London; Ftorafur (FF), Medex, Moscow; Fluorouracil (FU), Hoffmann-La-Roche, Basel; Oxauracyl (OXAUR), VUFB, Prague; Methotrexate (MTX), Lederle, Pearl River, N.Y.; TS-160, SPOFA, Prague; Vinblastine (VINBL), Gedeon Richter, Budapest; Vincristine (VINCR), Gedeon Richter, Budapest). Drug concentrations corresponded to those used for treatment in man (mg/g body wt converted to mg/ml medium).

Then $^3$H-thymidine ($^3$H-TdR 0·025 ml, 0·5 $\mu$Ci/ml, sp.act. 22 Ci/mmol UVVR, Prague) was added to give a final concentration of 2 $\mu$Ci/ml and the incubation was continued for a further hour. Controls without cytostatics were set up in each series of experiments. All tests were performed in duplicate. $^3$H-TdR incorporation into DNA was determined by liquid-scintillation counting. The effect of cytostatic drugs is expressed as a reduction in ct/min/$10^7$ cells in treated cells relative to the controls.

*Nuclear morphology in endometrial cancer undergoing progesterone treatment.*—Twenty patients with endometrial cancer who were given progesterone as a preliminary therapeutic measure have been investigated.

Tissue samples were obtained at the diagnostic curettage (A sample). All patients were then submitted to progesterone therapy (DEPO Provera, Upjohn S.A., Puurs, Belgium) i.m. on consecutive days to a total amount of 600 mg. On the 8th–10th day after progesterone therapy, several tissue samples (B sample) were obtained, either from curettage before intracavitary radium treatment, or at hysterectomy. All tissue specimens were fixed in methanol/formalin/acetic-acid mixture for 24 h, embedded in paraffin and 4 $\mu$m histological section were stained with haematoxylin and eosin. In each specimen (A or B) 150–200 tumour-cell nuclei were studied at random for measurement of the long and short axes, the ratio of the 2 axes being expressed as the "elongation". Secretory conversion of the endometrial cell population is morphologically expressed by shortening of the long axis of the nuclei, thus decreasing the elongation (Epifanova, 1971).

In all these specimens some aspects of cell kinetics were also studied using double labelling with $^3$H-TdR-$^{14}$C-TdR, the results of which are published separately (Siracký *et al.*, 1978).

### RESULTS AND DISCUSSION

### *Ploidy*

All the cases in our series were characterized before treatment by a wide range of ploidy, with modal chromosome numbers in the diploid or hyperdiploid range, which is characteristic of ascites tumour cell populations in ovarian cancer (Atkin, 1976; Koller, 1972).

"Number of mitoses studied" in Table I refers to samples studied before, during and after cytostatic treatment in sequence. Some of the cases are represented by only 2 samples (before and after treatment) due to inhibition of the ascites proliferation. "Modal chromosome number" refers to the pretreatment sample, and apart from case No. 11 (Fig. 2) no changes in modal chromosome number during cytostatic treatment were detected. In several of these cases, however, distinct changes in ploidy distribution

TABLE I.—*Survey of 12 advanced ovarian cancers with sequential chromosome studies of ascites tumour cells before, during and after cytostatic treatment*

| | | | | | | Ploidy distribution of mitoses | | | | | | | | | | | |
|---|---|---|---|---|---|---|---|---|---|---|---|---|---|---|---|---|---|
| | | | | | | Before treatment | | | | | | After treatment | | | | | |
| No. | Histology | Cytostatic treatment | No. of samples | No. of mitoses studied | Modal number | Near diplo | % | Near tetra | % | Higher ploidy | % | Near diplo | % | Near tetra | % | Higher ploidy | % |
| 1 | Adenoca | Leukeran +2 × 5 mg T.S. 160 i.p. | 3 | 107, 106, 70 | 59 | 79 | 73·8 | 5 | 4·6 | 23 | 21·6 | 43 | 61·4 | 7 | 10·1 | 20 | 28·5 |
| 2 | Adenoca | Mannogranol +1 × 5 mg TS-160 i.p. | 2 | 20, 70 | 50 | 19 | 95·0 | 1 | 5·0 | — | | 63 | 90·0 | 7 | 10·0 | — | |
| 3 | Papilloca | Cytembena | 5 | 98, 50, 36, 47, 112 | 51 | 82 | 83·7 | 10 | 10·2 | 6 | 6·1 | 85 | 75·9 | 27 | 24·1 | — | |
| 4 | Adenoca | Mannogranol +1 × 5 mg TS-160 i.p. | 2 | 70, 75 | 41 | 61 | 87·1 | 3 | 4·4 | 6 | 8·5 | 61 | 81·3 | 6 | 8·0 | 8 | 10·7 |
| 5 | Papilloca | Mannogranol +1 × 5 mg TS-160 i.p. | 2 | 50, 63 | 81 | 9 | 18·0 | 29 | 58·0 | 12 | 24·0 | 14 | 22·2 | 45 | 71·4 | 4 | 6·4 |
| 6 | Papilloca | Cytembena | 2 | 51,78 | 49 | 43 | 84·3 | 8 | 15·7 | — | | 61 | 78·2 | 17 | 21·8 | — | |
| *7 | Papilloca | Mannogranol +3 × 5 mg TS-160 i.p. | 4 | 98, 100, 101, 103 | 43 | 33 | 33·7 | 30 | 30·6 | 35 | 35·7 | 96 | 93·2 | 5 | 4·8 | 2 | 1·9 |
| 8 | Adenoca | Cytembena | 2 | 63, 68 | 56 | 55 | 87·3 | 8 | 12'7 | — | | 62 | 91·2 | 6 | 8·8 | — | |
| 9 | Adenoca | Mannogranol +1 × 5 mg TS-160 i.p. | 2 | 72, 54 | 54 | 69 | 95·8 | 3 | 4·2 | — | 50 | 50 | 92·6 | 4 | 7·4 | — | |
| 10 | Adenoca | Mannogranol +1 × 5 mg TS-160 i.p. | 2 | 72, 76 | 52 | 55 | 76·4 | 9 | 12·5 | 8 | 11·1 | 60 | 79·0 | 11 | 14·5 | 5 | 6·5 |
| †11 | Papilloca | Cytembena | 5 | 98, 68, 71, 81, 130 | 51 46 | 88 | 89·8 | 10 | 10·2 | — | | 126 | 96·9 | 4 | 3·1 | — | |
| 12 | Adenoca | TS-160 | 3 | 98, 101, 86 | 62 | 73 | 74·5 | 14 | 14·3 | 11 | 11·2 | 77 | 89·5 | 7 | 8·1 | 2 | 2·4 |

Total doses (all parenteral applications) during the course of the investigation (3–5 weeks): Leukeran—200 mg, Cytembena—6000 mg, Mannogranol—20·0 g, TS-160—25 mg. (+specified i.p. TS-160 doses).

* See Fig. 1. † See Fig. 2.

FIG. 1.—Ploidy distribution before (I) during (II, III) and after (IV) cytostatic treatment of tumour No. 7 in Table I.

FIG. 2.—Modal chromosome number before (I), during (II, III, IV) and after (V) cytostatic treatment of tumour No. 11 in Table I.

(cases 1, 3, 5, 7 and 12) during cytostatic treatment were evident. This type of chromosomal change seems to demonstrate selective overgrowth of cell clones, those resistant to the cytostatic treatment (Fig. 1 clearly demonstrates this type of change).

One spontaneous change with the age of the ascites is polyploidization (Koller, 1972) which is also to be expected as the result of damage to the mitotic process of cells by alkylating agents (Bishun, 1971; Nasjleti & Spencer, 1967). The ploidy distribution changes in our series are, however, characterized by decrease and/or disappearance of polyploid cells, which suggests increased sensitivity of polyploid clones to cytostatic drugs.

This form of sequential cytogenetic study during the follow-up of a patient (Eicke *et al.*, 1965; Mohr *et al.*, 1966; Siracký, 1969; Visfeldt & Lundwall, 1970) illustrates biological and cytogenetically expressed heterogeneity of the tumour-cell population in sensitivity to cytostatic agents, and contributes to a better understanding of the processes of cell selection in human tumours during treatment. There are, however, great difficulties in the interpretation of such analyses, since most of the cases with effusions are in an advanced stage, with metastatic dissemination and very poor prognosis.

### $^3$*H-TdR incorporation*

In most of the investigated cases, different parts of the same tumour, different metastatic nodes of the same patient, as well as the primary tumour and its ascitic form, differ significantly in their

TABLE II.—*Differences in drug sensitivity of samples from different areas within the same tumour (expressed as ct/min/10⁷ cells after uptake of [³H]-TdR)*

Ca colon

| Sample | Control ct/min | Control % | FU ct/min | FU % | MTX ct/min | MTX % | TS ct/min | TS % | VINCR ct/min | VINCR % |
|---|---|---|---|---|---|---|---|---|---|---|
| 1 | 309 | 100 | 289 | 93·5 | 90 | 29·1 | 77 | 24·9 | 212 | 68·6 |
| 2 | 384 | 100 | 256 | 66·6 | 80 | 20·8 | 44 | 11·4 | 52 | 13·5 |
| 3 | 421 | 100 | 312 | 74·1 | 487 | 115·7 | 95 | 22·6 | 7 | 1·6 |

Ca sigmae

| Sample | Control ct/min | Control % | FU ct/min | FU % | MTX ct/min | MTX % | TS ct/min | TS % | VINBL ct/min | VINBL % |
|---|---|---|---|---|---|---|---|---|---|---|
| 1 | 296 | 100 | 312 | 105·4 | 270 | 91·2 | 250 | 84·5 | 222 | 75·0 |
| 2 | 322 | 100 | 230 | 71·4 | 292 | 90·7 | 174 | 54·0 | 76 | 23·6 |

Ca ovary

| Sample | Control ct/min | Control % | FU ct/min | FU % | MTX ct/min | MTX % | TS ct/min | TS % |
|---|---|---|---|---|---|---|---|---|
| 1 | 457 | 100 | 403 | 88·2 | 312 | 68·2 | 125 | 27·3 |
| 2 | 397 | 100 | 305 | 76·8 | 370 | 93·2 | 231 | 58·2 |
| 3 | 524 | 100 | 400 | 76·3 | 305 | 58·2 | 96 | 18·3 |

Dose schedule in Tables II, III and IV:

| | |
|---|---|
| ALK— 0·2 mg/ml | OXAUR—0.01 mg/ml |
| FF— 2·0 mg/ml | TS— 0·1 mg/ml |
| FU— 0·5 mg/ml | VINBL— 0·1 mg/ml |
| MTX— 0·05 mg/ml | VINCR— 0·1 mg/ml |

TABLE III.—*Differences in drug sensitivity of samples from primary tumours and their metastases and/or between different metastatic nodes (Expressed as ct/min/10⁷ cells after uptake of [³H]-TdR)*

Advanced Ca ovary with metastatic dissemination

| Sample | Control ct/min | Control % | TS ct/min | TS % | MTX ct/min | MTX % | FU ct/min | FU % |
|---|---|---|---|---|---|---|---|---|
| Primary tumour 1 | 704 | 100 | 368 | 52·2 | 693 | 98·4 | 480 | 68·2 |
| Primary tumour 2 | 652 | 100 | 310 | 47·5 | 699 | 107·2 | 334 | 51·2 |
| Metastatic node | 786 | 100 | 249 | 31·7 | 522 | 66·4 | 449 | 57·1 |

Advanced Ca ovary—metastases omenti maioris

| Sample | Control ct/min | Control % | TS ct/min | TS % | MTX ct/min | MTX % | FU ct/min | FU % |
|---|---|---|---|---|---|---|---|---|
| Met. 1 | 801 | 100 | 165 | 20·6 | 1110 | 138·6 | 553 | 69·0 |
| Met. 2 | 661 | 100 | 157 | 23·7 | 367 | 55·5 | 221 | 33·4 |
| Met. 3 | 604 | 100 | 251 | 41·6 | 970 | 160·6 | 377 | 62·4 |

sensitivity to the same cytostatic treatment *in vitro* (Tables II, III, IV). These variations in tissue specimens might be explained by local nutritional conditions, by variations in the proliferation rate and/or by variations in the amount of necrosis in different parts of the tumour. These factors ought to be responsible for differences in the incorporation of the $^3$H-TdR in control specimens, but no large differences were found between controls. It can be assumed, therefore, that the differences in sensitivity to cytostatic treatment are due to the presence of 2 or more clones of tumour cells differing in their biological characteristics.

TABLE IV.—*Differences in drug sensitivity of primary tumours and their ascitic tumour-cell population (expressed as ct/min/$10^7$ cells after uptake of [$^3H$]-TdR)*

Advanced gastric cancer with ascites

| Sample | Control ct/min | Control % | ALK ct/min | ALK % | FU ct/min | FU % | MTX ct/min | MTX % | FF ct/min | FF % |
|---|---|---|---|---|---|---|---|---|---|---|
| Tumour | 399 | 100 | 432 | 108·2 | 213 | 53·4 | 195 | 48·9 | 384 | 96·2 |
| Ascites | 474 | 100 | 198 | 41·7 | 144 | 30·3 | 96 | 20·3 | 108 | 22·8 |

Advanced Ca ovary with ascites

| Sample | Control ct/min | Control % | FU ct/min | FU % | MTX ct/min | MTX % | TS ct/min | TS % | OXAUR ct/min | OXAUR % |
|---|---|---|---|---|---|---|---|---|---|---|
| Tumour | 378 | 100 | 371 | 92·2 | 668 | 176·7 | 211 | 55·8 | 309 | 81·8 |
| Ascites | 459 | 100 | 670 | 146·0 | 295 | 64·2 | 236 | 51·4 | 228 | 49·7 |

*N clear morphology*

Clinical experience has shown that endometrial cancers are hormone (progesterone) responsive in a considerable percentage of cases (Kelley & Baker, 1965; Smith *et al.*, 1966). Several studies have also demonstrated secretory conversion, mostly in highly differentiated cancers, manifesting itself by characteristic changes in nuclear morphology as a result of progesterone treatment (Hustin, 1976; Martinez-Manaton *et al.*, 1975). Secretory conversion implies loss of proliferative

TABLE V.—*% of cells from 20 endometrial cancers with mean length of nucleus <12 μm, before and after progesterone treatment*

| No. | Degree of differentiation | Before treatment | After treatment |
|---|---|---|---|
| 1 | H | 58·3 | 85·0 |
| 2† | H | 45·7 | 84·2 |
| 3 | H | 54·6 | 73·6 |
| 4 | H | 68·1 | 84·0 |
| 5 | H | 58·7 | 83·1 |
| 6 | H | 53·5 | 81·7 |
| 7 | H | 60·0 | 84·1 |
| 8 | M | 56·4 | 66·8 |
| 9 | M | 58·6 | 63·4 |
| 10 | M | 55·5 | 62·9 |
| 11 | M | 57·3 | 78·0 |
| 12 | M | 53·9 | 62·4 |
| 13 | M | 58·9 | 87·8 |
| 14* | M | 46·9 | 54·1 |
| 15 | M | 44·3 | 55·1 |
| 16* | M | 48·6 | 56·9 |
| 17 | P | 38·4 | 54·8 |
| 18* | P | 43·2 | 52·0 |
| 19* | P | 48·8 | 53·1 |
| 20* | P | 44·9 | 51·4 |

* Separation into 2 distinct cell populations as seen in Fig. 4.

† See Fig. 3.

H = high. M = Moderate. P = Poor.

12 μm was selected as the mean + s.d. for pretreatment samples.

FIG. 3.—Secretory conversion (as indicated by change in nuclear shape) of the tumour cells in a highly differentiated cancer (No. 2 in Table V). A = before and B = after progesterone treatment.

FIG. 4.—Separation of 2 distinct tumour-cell populations in their response to progesterone in a case of poorly differentiated cancer (No. 19 in Table V). A = before and B = after treatment.

capacity and might be a prelethal stage (Epifanova, 1971).

Most of the highly differentiated cancers in our series exhibited significant responses to progesterone, expressed as rising values of potential doubling time in post-treatment specimens (Siracký *et al.*, 1978) as well as showing secretory conversion of a significant fraction of the tumour-cell population (Table V, Fig. 3). In highly differentiated cancers of our series the ratio of long-short axis of nuclei changed from $1{\cdot}74 \pm 0{\cdot}52$ before to $1{\cdot}35 \pm 0{\cdot}41$ after progesterone treatment. The mean length of the long axis was $10{\cdot}38 \pm 2{\cdot}34$ μm before and $8{\cdot}69 \pm 2{\cdot}03$ μm after treatment.

Examples of the changes in nuclear morphology after progesterone treatment are shown in Figs 3 and 4. Table V presents the changes in percentage of nuclei $<12$ μm long in 20 different endometrial tumours.

In the group of moderately and poorly differentiated cancers, in spite of nearly unchanged values both in proliferation kinetics and nuclear morphology after progesterone treatment, a distinct fraction of the tumour-cell population undergoing secretory conversion could be detected by plotting the nuclear morphology parameters in nomograms (Fig. 4) which clearly demonstrated differences in response to progesterone treatment within the cell population of a single tumour. It appears, however, that recognition of 2 different types of cell population, as seen in Fig. 4B, would be possible only in tumours with about equal amounts of both cell types.

### REFERENCES

ATKIN, N. B. (1976) *Cytogenetic Aspects of Malignant Transformation.* Basel: Karger. p. 79.

BISHUN, N. P. (1971) The cytogenetic effect of cyclophosphamide on a Burkitt tumour cell line ($EB_4$) *in vitro. Mutat. Res.*, **11**, 258.

BISHUN, N. P., MILLS, J., LLOYD, N. & WILLIAMS, D. C. (1973) Chromosomal examination of various cell clones *in vitro* derived from a DMBA-induced male rat breast tumour. *Eur. J. Cancer*, **9**, 865.

EICKE, J., EMMINGER, A., STRAUSS, C., MOHR, M. & WRBA, H. (1965) Cytogenetisch-karyologische Studien an Klinisch behandelten gynäkologischen Tumoren. *Z. Krebsforsch.*, **67**, 205.

EPIFANOVA, O. I. (1971) Effects of hormone on the cell cycle. In *The Cell Cycle and Cancer.* Ed. R. Baserga. New York: Dekker. p. 160.

HAKANSSON, L. & TROPÉ, C. (1974) On the presence within tumours of clones that differ in sensitivity to cytostatic drugs. *Acta Pathol. Microbiol. Scand.*, **82**, 35.

HUSTIN, J. (1976) Morphology and DNA content of endometrial cancer nuclei under progesterone treatment. *Acta Cytol.* (*Baltimore*), **20**, 556.

KELLEY, R. M. & BAKER, W. H. (1965) The role of progesterone in human endometrial cancer. *Cancer Res.*, **25**, 1190.

KOLLER, P. C. (1972) *The Role of Chromosomes in Cancer Biology.* Berlin, New York: Springer. p. 50.

MARTINEZ-MANATON, J., MASQUEO, M., AZNAR, R., PHARRIS, B. B. & ZAFFARONI, A. (1975) Endometrial morphology in women exposed to uterine system releasing progesterone. *Am. J. Obstet. Gynecol.*, **121**, 175.

MIAN, N., COROCU, D. M. & NUTMAN, C. A. (1974) Glycosidase heterogeneity among dimethylhydrazine induced rat breast tumours. *Eur. J. Cancer*, **9**, 865.

MITELMAN, F. (1971) The chromosomes of fifty Rous rat sarcomas. *Hereditas*, **69**, 155.

MOHR, U., EMMINGER, A., EICKE, J. & STRAUSS, C. (1966) Cytologische Untersuchungen an Tumorzellen in Exudaten von Patientinen mit gynäkologischen Tumoren. *Gynaecologia*, **161**, 49.

NASJLETI, C. E. & SPENCER, H. H. (1967) Chromosome polyploidisation in human leukocyte treated with streptonigrin and cyclophosphamide. *Cancer*, **20**, 31.

PÁLYI, J., OLAH, F. & SUGAR, J. (1977) Drug sensitivity studies on clonal cell lines isolated from heteroploid tumour cell population. I. Dose response of clones growing in monolayer cultures. *Int. J. Cancer*, **19**, 859.

SIRACKÝ, J. (1969) Ploidy studies in ovarian cancer during cytostatic treatment. *Neoplasma*, **16**, 427.

SIRACKÝ, J., SCHREINER, P., SIRACKÁ, E. & MAT'OŠKA, J. (1978) Cell proliferation kinetics and nuclear morphology in endometrial cancer under progesteron treatment. *Neoplasma*, **25**, 535.

SMITH, J. P., RUTLEDGE, F. & SOFFAR, S. W. (1966) Progestins in the treatment of patients with endometrial carcinoma. *Am. J. Obstet. Gynecol.*, **94**, 977.

TROPÉ, C. & HAKANSSON, L. (1974) An *in vitro* study of cytostatic drug effect on the DNA synthesis in methylcholanthrene induced mouse sarcomas. *Acta Pathol. Microbiol. Scand.*, **82**, 189.

TROPÉ, C., HAKANSSON, L. & DENCKER, H. (1975) Heterogeneity of human adenocarcinomas of the colon and the stomach as regards sensitivity to cytostatic drugs. *Neoplasma*, **22**, 423.

VISFELDT, J. & LUNDWALL, F. (1970) Alteration of karyotypic profiles in human cancerous effusions following treatment with antineoplastic drug. *Acta Pathol. Microbiol. Scand.*, **78**, 551.

WHISSON, M. E. & SIRACKÝ, J. (1969) Increased sensitivity of a plasma cell tumour to TEM on conversion to the ascitic form: relationship to karyotype and cycle kinetics. *Neoplasma*, **16**. 347.